# Evaluation of Cardiac Function in Young Mdx Mice Using MRI with Feature Tracking and Self-Gated Magnetic Resonance Cine Imaging

**DOI:** 10.3390/diagnostics13081472

**Published:** 2023-04-19

**Authors:** Junpei Ueda, Shigeyoshi Saito

**Affiliations:** 1 Department of Medical Physics and Engineering, Division of Health Sciences, Osaka University Graduate School of Medicine, Suita 560-0871, Osaka, Japan; 2 Department of Advanced Medical Technologies, National Cardiovascular and Cerebral Research Center, Suita 564-8565, Osaka, Japan

**Keywords:** cardiac function, young mdx mice, feature tracking, self-gated magnetic resonance cine imaging

## Abstract

This study aimed to evaluate cardiac function in a young mouse model of Duchenne muscular dystrophy (mdx) using cardiac magnetic resonance imaging (MRI) with feature tracking and self-gated magnetic resonance cine imaging. Cardiac function was evaluated in mdx and control mice (C57BL/6JJmsSlc mice) at 8 and 12 weeks of age. Preclinical 7-T MRI was used to capture short-axis, longitudinal two-chamber view and longitudinal four-chamber view cine images of mdx and control mice. Strain values were measured and evaluated from cine images acquired using the feature tracking method. The left ventricular ejection fraction was significantly less (*p* < 0.01 each) in the mdx group at both 8 (control, 56.6 ± 2.3% mdx, 47.2 ± 7.4%) and 12 weeks (control, 53.9 ± 3.3% mdx, 44.1 ± 2.7%). In the strain analysis, all strain value peaks were significantly less in mdx mice, except for the longitudinal strain of the four-chamber view at both 8 and 12 weeks of age. Strain analysis with feature tracking and self-gated magnetic resonance cine imaging is useful for assessing cardiac function in young mdx mice.

## 1. Introduction

Duchenne muscular dystrophy (DMD) is an X-linked severe progressive muscle wasting disease caused by a deficiency in dystrophin protein [1,2,3]. A dystrophin deficiency leads to dilated cardiomyopathy (DCM), which may occur during adolescence [4,5]. DCM is a myocardial disease characterized by left ventricular or biventricular diastolic and systolic dysfunction in the absence of sufficient pressure/volume loading or coronary artery disease [6,7]. The C57BL/10-mdx (mdx) mouse model of human muscular dystrophy carries a mutation in exon 23 of the Xp21 region of the genome, resulting in the loss of dystrophin protein expression [8]. The DMD research has been conducted using the mdx model, which has a mutation in the dystrophin gene itself like DMD patients [9,10]. DCM is considered to be the main cause of death in DMD, and the mechanism of DCM is not yet fully elucidated [11]. Therefore, it is very important to elucidate the early pathogenesis of DCM by young mdx mice.

Cardiovascular magnetic resonance (CMR) imaging is used to diagnose various cardiac diseases, and its usefulness in DCM has been reported [12,13,14]. The evaluation of cardiac function using CMR in mice is as reproducible as it is in humans [15] and has also been used to evaluate the pathogenesis of DCM [16,17]. In CMR, the evaluation of cardiac function can be quantified, including the left ventricular ejection fraction and strain analysis. In recent years, feature-tracking methods have attracted attention in addition to conventional methods, such as tagging and harmonic phase methods in strain analysis. The feature-tracking method can calculate strain values from standard cine CMR images without additional CMR images [18,19]. The cardiac function of mdx mice has been evaluated with CMR [20,21]; however, these studies evaluated older mice or calculated strain values without using the feature tracking method.

This study aimed to assess cardiac function in young mdx mice, including strain assessment using the feature tracking method, and to observe changes in cardiac function in mdx mice.

## 2. Materials and Methods

### 2.1. Animal Preparation

All experimental protocols were approved by the Research Ethics Committee of Osaka University. All experimental procedures involving animals and their care were performed in accordance with the University Guidelines for Animal Experimentation and the National Institutes of Health Guide for the Care and Use of Laboratory Animals. Animal experiments were performed on male C57BL/6JJmsSlc mice (control mice) 8–12 weeks old purchased from Japan SLC (Hamamatsu, Japan) and C57BL/10ScSn-Dmdmdx/JicJcl mice (mdx mice) purchased from CLEA Japan, Inc. (Tokyo, Japan). All mice were housed in a controlled vivarium environment (24 °C; 12:12 h light:dark cycle) and fed a standard pellet diet with water ad libitum. MRI experiments were performed at 2 time points at 8 weeks of age (10 control mice and 10 mdx mice) and 12 weeks of age (10 control mice and 10 mdx mice).

### 2.2. Magnetic Resonance Imaging

MR images of animal hearts were acquired using a horizontal 7-T scanner (PharmaScan 70/16 US; Bruker Biospin; Ettlingen, Germany) equipped with a volume coil with an inner diameter of 30 mm. For MRI, the mice were positioned with their mouth in a stereotaxic frame to prevent movement in a prone position during acquisition. The mice were maintained at a body temperature of 36.5 °C, with water flow regulated, and continuously monitored using a physiological monitoring system (SA Instruments Inc., Stony Brook, NY, USA). All MRI experiments on mice were performed under general anesthesia with 1.0–2.0% isoflurane in an air–oxygen mixture (Abbott Laboratories; Abbott Park, IL, USA) administered through a mask covering the nose and mouth of the mice. 

Short-axis, long-axis two-chamber, and long-axis four-chamber images were obtained using fast low-angle shots (FLASH) with a self-gated magnetic resonance cine imaging system (). For short-axis images, the following parameters were used: repetition time (TR)/echo time (TE) = 44.5/2.5 ms, flip angle = 25°, movie frames = 14 frames per cardiac cycle, field of view (FOV) = 25.6 × 25.6 mm, acquisition time = 23:43 min, in-plane resolution per pixel = 133 µm, matrix = 192 × 192, number of excitations (NEX) = 300, oversampling = 1, and five concomitant slices covering the whole heart from the apex to the base. For the long-axis two- and four-chamber views, the parameters were TR/TE = 6.5/3.1 ms, flip angle = 10°, movie frames = 14 frames/s, FOV = 25.6 mm × 25.6 mm, acquisition time = 3:52 min, in-plane resolution per pixel = 133 µm, matrix = 192 × 192, NEX = 1, and oversampling = 250. The total scan time per animal was approximately 40 min. 

### 2.3. MRI Data Analysis

The cardiac MR images were analyzed using cvi42 software (Circle Cardiovascular Imaging, Calgary, AB, Canada). The borders of the epicardium and endocardium were outlined manually on the short-axis images, two- chamber long-axis images and four-chamber long-axis images at both end-diastolic phase and end-systolic phase. The left ventricular end-diastolic volume (LVEDV), left ventricular end-systolic volume (LVESV), left ventricular stroke volume (LVSV), left ventricular ejection fraction (LVEF), and left ventricular mass (LVM) were calculated from cine images. All values were calculated automatically by Cvi42. In addition, strain analysis was performed using feature tracking. For myocardial strain analysis, the global radial strain (RS), global circumferential strain (CS), and longitudinal strain (LS) values were calculated. LS was analyzed from the 2- and 4-chamber long-axis views. The peak value of each strain was used for statistical evaluation.

### 2.4. Statistical Analysis

The LVESV, LVEDV, LVSV, LVEF, LVM, and each strain value calculated from the MR images are presented as the mean ± standard deviation. All statistical analyses were performed using Prism, version 9 (GraphPad Software; San Diego, CA, USA). Differences were compared using a one-way analysis of variance and Tukey’s multiple comparison test. Statistical significance was set at *p* < 0.05 (* *p* < 0.05, ** *p* < 0.01, *** *p* < 0.001).

### 2.5. Histological Analysis and Immunostaining

After completion of the MRI at 12 weeks of age, the mice were sacrificed, and their hearts were removed and fixed in formalin. Fixed hearts were sliced in the direction of the short axis of the left ventricle. Heart specimens were embedded in paraffin and sectioned at 5 μm; some sections were stained with hematoxylin and eosin (H&E), and some sections were immunostained. The stained tissues were observed using an optical microscope (Keyence Corporation; Osaka, Japan). H&E staining was performed by immersing samples in a hematoxylin solution (5 min) and alcohol–eosin staining solution (3 min). H&E-stained tissues were dehydrated six times with 100% alcohol and then permeabilized using xylene. Immunostaining was performed using the enzyme–antibody method. Dystrophin rabbit polyclonal antibodies (12715-1-P; Proteintech Group, Inc., Rosemont, IL, USA) were used as the primary antibody; the samples were incubated for 1 h. After washing three times for 5 min each, the samples were incubated with the EnVision+ horseradish peroxidase-conjugated anti-rabbit secondary antibodies (K4003; Agilent Technologies, Inc., Santa Clara, CA, USA) for 30 min.

## 3. Results

### 3.1. Animals’ Characteristics

The characteristics of the animals are summarized in Table 1. No significant difference in body weight or respiratory rate was found between the control and mdx groups of the same age. However, both the 8- and 12-week-old groups showed significant differences between the groups in heart rate.

### 3.2. Visual Evaluation of Cine Images of Short Axis, Two-Chamber, and Four-Chamber View

**Figure 1:**
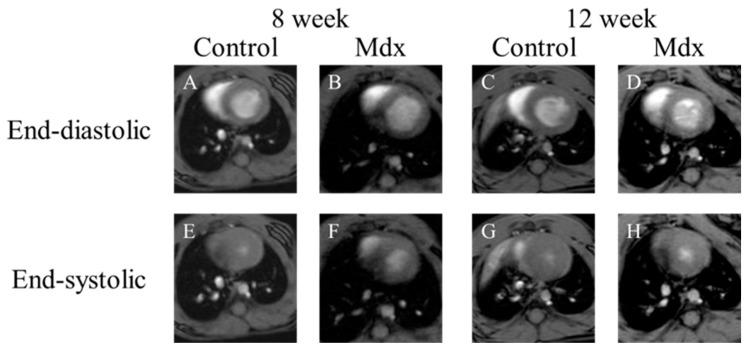
Representative short-axis view cine magnetic resonance images: (**A**,**E**) 8-week-old control mouse, (**B**,**F**) 8-week-old mdx mouse, (**C**,**G**) 12-week-old control, (**D**,**H**) 12-week-old mdx mouse. (**A**–**D**) The end-diastolic phase of a mouse heart; (**E**–**H**) the end-systolic phase of a mouse heart.

**Figure 2:**
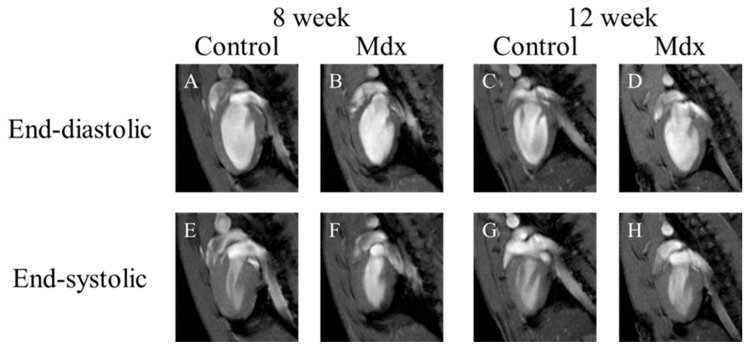
Representative two-chamber view cine magnetic resonance images: (**A**,**E**) 8-week-old control mouse, (**B**,**F**) 8-week-old mdx mouse, (**C**,**G**) 12-week-old control, (**D**,**H**) 12-week-old mdx mouse. (**A**–**D**) The end-diastolic phase of a mouse heart; (**E**–**H**) the end-systolic phase of a mouse heart.

**Figure 3:**
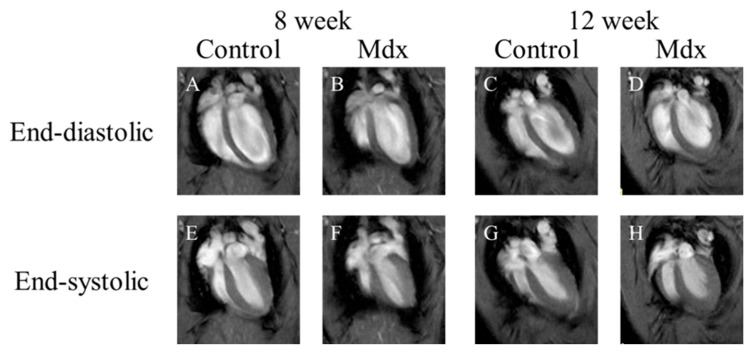
Representative four-chamber view cine magnetic resonance images: (**A**,**E**) 8-week-old control mouse, (**B**,**F**) 8-week-old mdx mouse, (**C**,**G**) 12-week-old control, (**D**,**H**) 12-week-old mdx mouse. (**A**–**D**) The end-diastolic phase of a mouse heart; (**E**–**H**) the end-systolic phase of a mouse heart.

In short-axis cine images and the two-chamber view cine images, contraction and expansion of the entire myocardium were observed in the control group at 8 and 12 weeks of age. For LVEDV, no significant difference was observed between the control and mdx groups (Figure 1B,D and Figure 2B,D). However, the myocardial contraction was weaker in the mdx group than in the control group (Figure 1F,H and Figure 2F,H). In the four-chamber view cine images, contraction and expansion of the entire myocardium were observed in the control group at 8 and 12 weeks of age. However, no differences in shrinkage were observed in four-chamber views (Figure 3F,H).

### 3.3. Cardiac Function in 8- and 12-Week-Old Mdx Mice vs. Age-Matched Controls

**Figure 4:**
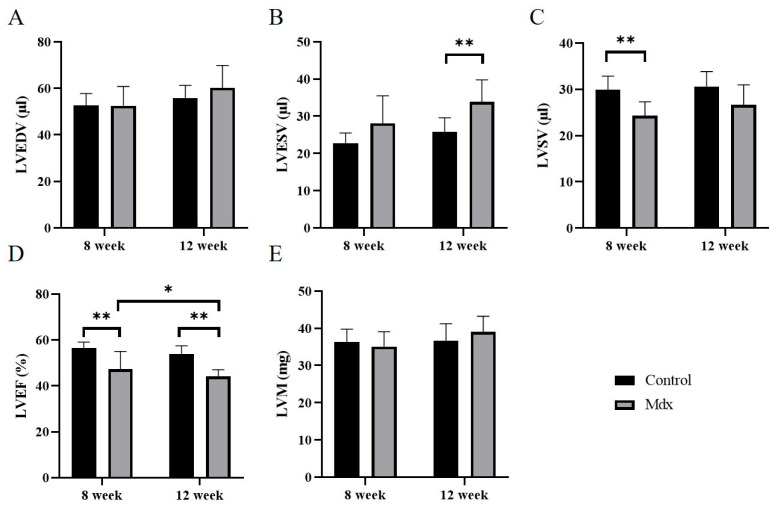
Graphs quantifying LVEDV (**A**), LVESV (**B**), LVSV (**C**), LVEF (**D**), and LVM (**E**) in the 8-week-old control mice, 8-week-old mdx mice, 12-week-old control mice, and 12-week-old mdx mice. LVEDV, left ventricular end-diastolic volume; LVESV, left ventricular end-systolic volume; LVSV, left ventricular stroke volume; LVEF, left ventricular ejection fraction; LVM, left ventricular mass. * *p* < 0.05, ** *p* < 0.01, respectively.

Regarding the LVEDV (Figure 4A), no significant difference was found between the control and mdx groups at 8 weeks old (control, 52.6 ± 4.9 μL; mdx, 52.5 ± 7.9 μL) and 12 weeks old (control, 55.9 ± 5.1 μL; mdx, 60.3 ± 9.1 μL). Regarding the LVESV (Figure 4B), no significant difference was found between the control and mdx groups at 8 weeks old (control, 22.8 ± 2.6 μL; mdx, 28.1 ± 7.0 μL). However, at 12 weeks old, LVESV was significantly less in the mdx group compared to the control group (control, 25.8 ± 3.6 μL; mdx, 33.9 ± 5.6 μL, *p* < 0.01). Regarding the LVSV (Figure 4C), a significant difference was identified between the control and mdx groups at 8 weeks old (control, 29.9 ± 2.8 μL; mdx, 24.3 ± 2.8 μL, *p* < 0.01). However, at 12 weeks old, no significant difference was found between the control and mdx groups (control, 30.6 ± 3.1 μL; mdx, 26.6 ± 4.2 μL). Regarding the LVEF (Figure 4D), a significant difference was identified between the control and mdx groups at 8 weeks (control, 56.6 ± 2.3%; mdx, 47.2 ± 7.4%, *p* < 0.01) and 12 weeks (control, 53.9 ± 3.3%; mdx, 44.1 ± 2.7%, *p* < 0.01). A significant difference was also identified between the 8- and 12-week-old mice in the mdx group (*p* < 0.05). Regarding the LVM (Figure 4E), no significant difference was found between the control and mdx groups at 8 weeks old (control, 36.3 ± 3.3 mg; mdx, 35.1 ± 3.8 mg) and 12 weeks old (control, 36.7 ± 4.3 mg; mdx, 39.0 ± 4.0 mg).

### 3.4. Strain Analysis by Feature-Tracking Method in 8- and 12-Week-Old Mdx Mice vs. Age-Matched Controls

**Figure 5:**
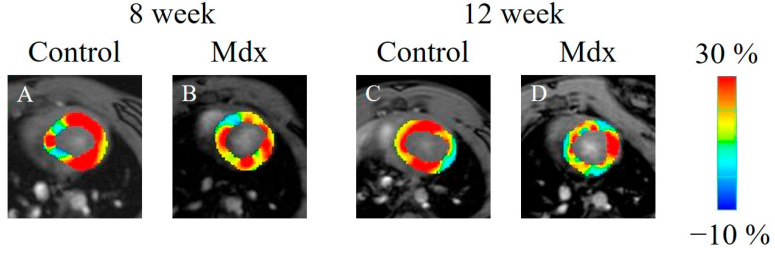
Radial strain-encoded functional magnetic resonance imaging of the end-systolic left ventricle: (**A**) 8-week-old control mouse, (**B**) 8-week-old mdx mouse, (**C**) 12-week-old control mouse, (**D**) 12-week-old mdx mouse. The color bar shows the scale of the strain based on the end-diastolic left ventricle, with maximum contraction shown in red and minimum contraction in blue.

**Figure 6:**
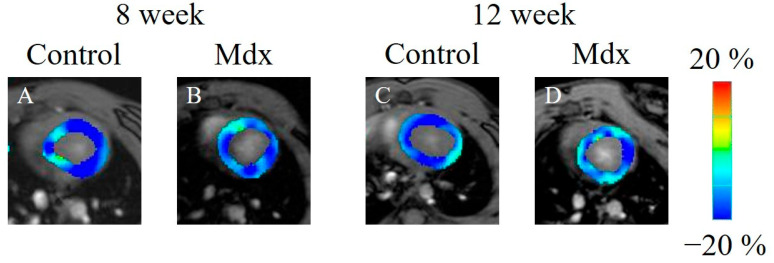
Circumferential strain-encoded functional magnetic resonance imaging of the end-systolic left ventricle: (**A**) 8-week-old control mouse, (**B**) 8-week-old mdx mouse, (**C**) 12-week-old control mouse, (**D**) 12-week-old mdx mouse. The color bar shows the scale of the strain based on the end-diastolic left ventricle, with maximum contraction shown in blue and minimum contraction in red.

**Figure 7:**
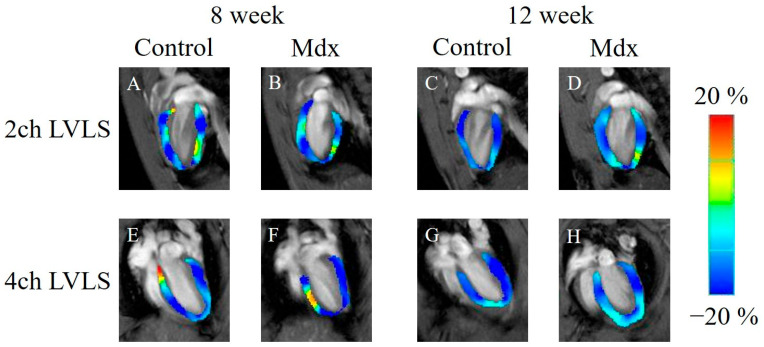
Longitudinal strain-encoded functional magnetic resonance imaging of the end-systolic left ventricle: (**A**,**E**) 8-week-old control mouse, (**B**,**F**) 8-week-old mdx mouse, (**C**,**G**) 12-week-old control mouse, (**D**,**H**) 12-week-old mdx mouse. The color bar shows the scale of the strain based on the end-diastolic left ventricle, with maximum contraction shown in blue and minimum contraction in red. 2ch LVLS: two-chamber-view longitudinal strain of left ventricle, 4ch LVLS: four-chamber-view longitudinal strain of left ventricle.

**Figure 8:**
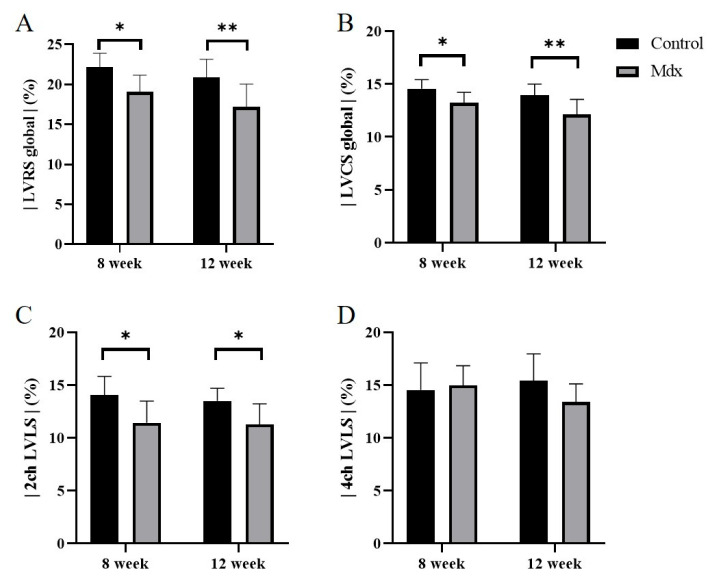
Graphs quantifying the strain analysis in the 8-week-old control mice, 8-week-old mdx mice, 12-week-old control mice, and 12-week-old mdx mice. (**A**) Global radial strain of left ventricle (LvRS global), (**B**) global circumferential strain of left ventricle (LVCS global), (**C**) two-chamber-view longitudinal strain of left ventricle (2ch-LVLS), (**D**) four-chamber-view longitudinal strain of left ventricle (4ch-LVLS). * *p* < 0.05, ** *p* < 0.01, respectively.

In the strain analysis of the 2ch-LS and short-axis images, the peak strain values were lower in the mdx group compared with the control group. The areas of reduction in strain values were diffuse, and no regularity was apparent (Figure 5, Figure 6 and Figure 7). According to statistical analysis of strain values, the global radial strain of the left ventricle was significantly less in the mdx group compared to the control group at 8 weeks old (control, 22.2 ± 1.7%; mdx, 19.0 ± 2.0%, *p* < 0.05) and 12 weeks old (control, 20.9 ± 2.2%; mdx, 17.2 ± 2.7%, *p* < 0.01; Figure 8A). The global circumferential strain of the left ventricle was significantly less in the mdx group compared to the control group at 8 weeks old (control, −14.6 ± 0.8%; mdx, −13.2 ± 0.9%, *p* < 0.05) and 12 weeks old (control, −13.9 ± 1.0%; mdx, −12.1 ± 1.4%, *p* < 0.01; Figure 8B). The two-chamber-view longitudinal strain of the left ventricle (2ch-LVLS) was significantly less in the mdx group compared with the control group at 8 weeks old (control, −14.1 ± 1.7%; mdx, −11.4 ± 2.0%, *p* < 0.05) and 12 weeks old (control, −13.5 ± 1.1%; mdx, −11.3 ± 1.9%, *p* < 0.05; Figure 8C). Regarding the strain values of the global four-chamber-view longitudinal strain of the left ventricle (4ch-LVLS), no significant difference was found between the control and mdx groups at 8 weeks (control, −14.5 ± 2.4%; mdx, −15.0 ± 1.8%) and 12 weeks (control, −15.4 ± 2.4%; mdx, −13.4 ± 1.6%; Figure 8D). 

### 3.5. H&E Staining and Immunostaining of Myocardium Tissue

**Figure 9:**
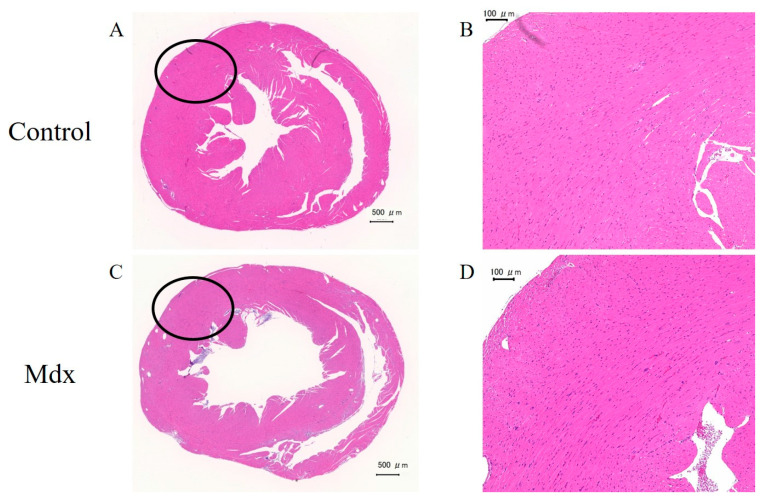
Heart sections stained with hematoxylin and eosin from (**A**,**B**) control and (**C**,**D**) mdx mice. (**B**,**D**) Magnified images of the black-circle regions. Scale bars represent 500 µm (**A**,**C**) and 100 µm (**B**,**D**).

**Figure 10:**
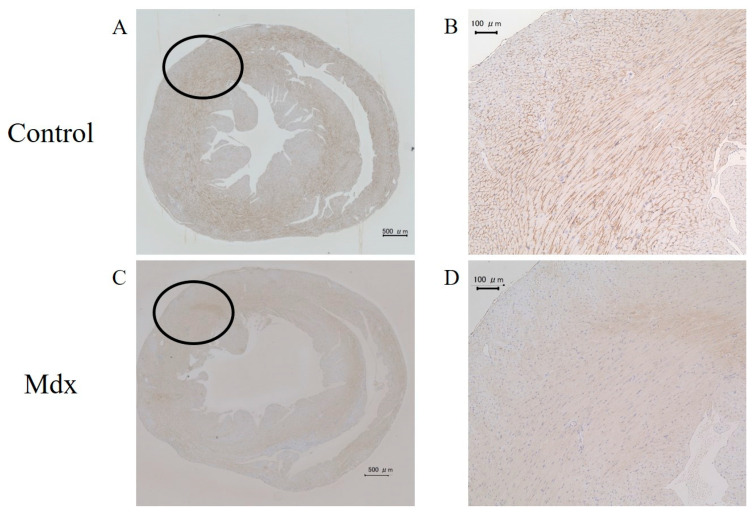
Heart sections stained with antibodies against dystrophin from (**A**,**B**) control and (**C**,**D**) mdx mice. (**B**,**D**) Magnified images of the black-circle regions. Scale bars represent 500 µm (**A**,**C**) and 100 µm (**B**,**D**).

In the H&E staining results, no differences were observed between the mdx and control groups (Figure 9). However, staining with antibodies against dystrophin showed differences between the mdx and control groups (Figure 10). Staining of the intercellular matrix was observed in the control group (Figure 10A,B) but not in the mdx group (Figure 10C,D).

## 4. Discussion

In this study, myocardial cine images of mdx mice were acquired using 7-T MRI at 8 and 12 weeks of age to evaluate cardiac function and myocardial strain values. No significant difference was found in the left ventricular end-diastolic volume at 8 and 12 weeks of age. However, both at 8 and 12 weeks of age, the left ventricular ejection fraction showed a decreasing trend in the mdx group compared with the control group. In strain analysis, all peak strain values showed a decreasing trend, except for 4ch-LS at 8 and 12 weeks of age. Previous studies using CMR have reported lower cardiac function in older mdx mice than we found in our study [20,21]. However, these previous studies did not use the feature tracking, ECG, and self-gated magnetic resonance cine imaging used in our study. The present study’s novel strain analysis, ECG, and respiratory-gated methods suggest that young mdx mice have less cardiac function.

Compared to the conventional tagging method, the feature-tracking method does not require additional imaging and can be performed from cine images for strain analysis. Self-gated magnetic resonance cine imaging allows ECG and respiration gating without the need to wear monitoring equipment. Therefore, in addition to the advantage of being able to detect a decline in cardiac function that could not be detected by conventional methods, these novel methods also have the significant advantage of reducing examination time.

Previous studies have reported no change in cardiac function until older age [21,22,23,24]. At the age of 8 months, the contractile and diastolic functions of the left ventricular myocardium decrease, thereby decreasing the left ventricular ejection fraction [20]. However, a previous study showed that CMR taken at a high temporal resolution showed reduced cardiac function in 3-month-old mdx mice [25]. Although the temporal resolution differs from this previous study, our study and other studies [20,26] suggest a decline in cardiac function in young mdx mice. Consistent with a previous study [20], immunostaining showed no staining of the extracellular matrix of cardiomyocytes in mdx mice compared to the control group (Figure 10). Hence, immunostaining showed a difference in cardiomyocytes between the mdx and control groups, and CMR also detected less cardiac function in mdx group. However, the CMR results did not show ventricular enlargement, which is a symptom of dilated cardiomyopathy. In addition, no areas of necrosis were found in the cardiomyocytes of mdx mice in H&E staining unlike previous studies [20]. Thus, our study suggests that mdx mice have impaired cardiac function at a stage prior to DCM diagnosis.

This study had limitations. First, by evaluating cardiac function in 8- and 12-week-old mdx mice, this study demonstrated this methodology’s utility for detecting changes in cardiac function in young mdx mice. However, cardiac function after 12 weeks of age has not been evaluated. Evaluation of cardiac function after 12 weeks of age would provide more details on the transition of cardiac function in mdx mice during aging. Analysis of cardiac function after 12 weeks may reveal some changes in LVEDV, which did not differ significantly in this study. A more detailed cardiac function transition may allow better investigation of cardiac function treatment in mdx mice [27,28], including different genotypes [29,30]. Second, to demonstrate the usefulness of the present method, we evaluated the cardiac function of mdx mice using feature tracking and self-gated magnetic resonance cine imaging and compared them with previous studies. By comparing this study with conventional ECG synchronization and tagging methods, it may be possible to demonstrate the usefulness of the method used in this study in more detail. Third, it is considered that there is an effect of sex difference on the decrease in cardiac function caused by DMD [31]. Since only male mice were used in this study, it is necessary to examine the effects of sex differences on the results in the future. To make evaluating cardiac function in mdx mice more accurate, further studies are needed to evaluate longer-term cardiac function and assess cardiac function using various methods.

## 5. Conclusions

This study showed that strain analysis with feature tracking and self-gated magnetic resonance cine imaging is useful for assessing cardiac function in young mdx mice. Using these methods, we demonstrated that young mdx mice decline in cardiac function. 

## Figures and Tables

**Table 1:** Animal characteristics.

	8 Week		12 Week	
	Control (*n* = 10)	Mdx (*n* = 10)	Control (*n* = 10)	Mdx (*n* = 10)
Body weight (g)	25.6 ± 1.3	26.2 ± 1.2	28.3 ± 1.1	29.3 ± 1.6
Heart rate (bpm)	321 ± 32	275± 23 **	334 ± 35	277 ± 22 **
Respiratory rate (brpm)	73.4 ± 3.4	65.5 ± 6.6	76.2 ± 4.6	70 ± 9.4

** *p* < 0.01 between mdx mice and age-matched controls.

## Data Availability

The data presented in this study are available on request from the corresponding author.
